# Role of Stingray (*Himantura signifier*) Non-Protein Nitrogenous Fraction on the Oxidative Stability of Lipid and Myoglobin

**DOI:** 10.3390/foods12020274

**Published:** 2023-01-07

**Authors:** Worawan Panpipat, Jutaporn Limsuwanmanee, Ling-Zhi Cheong, Manat Chaijan

**Affiliations:** 1 Food Technology and Innovation Research Center of Excellence, School of Agricultural Technology and Food Industry, Walailak University, Nakhon Si Thammarat 80160, Thailand; 2 School of Agriculture and Food, Faculty of Veterinary and Agricultural Sciences, The University of Melbourne, Parkville, VIC 3010, Australia

**Keywords:** lipid oxidation, myoglobin, antioxidant, non-protein nitrogen, fish

## Abstract

Non-protein nitrogen (NPN) is abundant in stingray (*Himantura signifier*) muscle, which also has in vitro antioxidant activity. In this study, NPN from stingray muscle was further investigated for its antioxidant properties in lecithin liposome and oxymyoglobin model systems to validate its protective impact against lipid and myoglobin oxidations during storage for 120 min at various temperatures (4, 25, and 60 °C). NPN solution (10 ppm nitrogen) was added to the lecithin liposome system at different concentrations (0, 0.5, 1, 5, and 10% (*v*/*v*)) to investigate its effects on lipid stability by measuring the conjugated diene (CD), peroxide value (PV), and thiobarbituric acid reactive substances (TBARS) contents. In the oxymyoglobin system, NPN solution (10 ppm nitrogen) was also added at different concentrations (0, 0.5, 1, 5, and 10% (*v*/*v*)) to the oxymyoglobin solution in order to examine its effect on the stability of myoglobin by determining the contents of oxymyoglobin, metmyoglobin, and protein carbonyl. According to the findings, in all NPN concentrations, the system incubated at 4 °C had the lowest levels of lipid oxidation as measured by CD, PV, and TBARS values, and the lowest levels of myoglobin oxidation. At all incubating temperatures, the oxymyoglobin and lipid oxidation of all model systems tended to rise with the lengthening of the incubation duration. With the addition of 5% NPN, however, the lowest CD, PV, TBARS, oxymyoglobin oxidation, metmyoglobin formation, and protein carbonyl content were all observable, and the remarkable result was discovered during incubation at 4 °C. The results indicate that stingray NPN, especially at 5%, can be used to delay lipid and myoglobin oxidation, particularly at 4 °C. In order to prolong the shelf life of products with dark-fleshed fish and red meat, stingray NPN might be used as an alternative antioxidant to delay the oxidation of lipid and myoglobin during cold chain storage.

## 1. Introduction

Nitrogenous substances are key components of fish meat, influencing both its nutritive benefits and its sensory attributes. Protein and non-protein nitrogen (NPN) molecules are the main nitrogenous components found in fish flesh [1,2]. The content of NPN compounds in marine animal meat varies depending on the species, environment, and growth stage, as well as the condition of freshness after harvesting. NPN accounts for 9–15% of total N in white-fleshed fish, 16–18% in clupeides, and up to 55% in some sharks. In average, ordinary meat has more NPN than dark meat in some species such as mackerel (*Auxis thazard*) and catfish (*Clarias macrocephalus*) [3]. NPN compound distribution varies by taxonomic family [4]. Approximately 95% of the total content of NPN in marine fish muscle is made up of free amino acids, peptides, urea, trimethylamineoxide (TMAO) and its breakdown products, guanidine substances, nucleotides and their postmortem metabolites, and betaines [5]. Carnosine, anserine, and balenine are the most common peptides detected in meat and fish [6]. Endogenous proteolytic activity produces oligopeptides, short-sized peptides, and free amino acids, which can be released in fish during storage and consumed as food ingredients [7]. Endogenous peptides from Pacific hake (*Merluccius productus*) [8], European sea bass (*Dicentrarchus labrax*) [7], and farmed hybrid catfish (*Clarias macrocephalus* × *Clarias gariepinus*) [9] have demonstrated interesting antioxidant effects. Chaijan et al. [9] discovered endogenous peptides with antioxidant properties in the muscle of farmed hybrid catfish. These peptides ranged in length from 8 to 24 amino acid residues, with varied hydrophobic amino acid composition and unique sequences.

Multiple studies have shown that water-soluble NPN compounds, such as nucleotides, polyamines, histidine-containing dipeptides (carnosine, anserine, and balenine), and glutathione tripeptides, have a high potential for usage as natural food antioxidants [2,10,11,12,13,14]. According to Elias et al. [15], peptides containing a large amount of oxidatively labile amino acid residues (e.g., cysteine, tryptophan, and methionine) can prevent lipid oxidation. According to Sasaki et al. [16], tissue from an underutilized salmon by-product has a multicomponent antioxidant defense system such as polyamines, spermine, and spermidine. Furthermore, Løvaas [12] found that spermine prevents fish oil oxidation more effectively than α-tocopherol, butylated hydroxyanisole, tert-butylhydroquinone, and ascorbyl palmitate. An extracted aqueous fraction or press juice from white-fleshed fish such as cod (*Gadhus morhua*), haddock (*Melanogrammus aeglefinus*), dab (*Hippoglossoides plattessoides*), and winter flounder (*Pseudopleuronectes americanus*) and dark-fleshed fish such as herring (*Clupea harengus*) were found to have strong inhibitory efficacy against lipid oxidation of washed cod mince membranes [17,18]. Press juice from herring has also been employed to postpone lipid oxidation in frozen herring fillets [19]. Additionally, fish discards from species such as greater weever (*Trachinus draco*), painted cober (*Serranus scriba*), flying gurnard (*Dactylopterus volitans*), Atlantic bonito (*Sarda sarda*), common pandora (*Pagellus erythinus*), merling (*Merlangius merlangus*), and annular seabream (*Diplodus annularis*) have the potential to play a role as raw materials for producing press juice with antioxidant activity [20].

Limsuwanmanee et al. [2] reported the NPN levels in entire muscle from tropical fish, including threadfin bream (*Nemipterus bleekeri*), Indian mackerel (*Rastrelligar kanagurta*), tilapia *(Oreochromis niloticus*), and stingray (*Himantura signifier*) at the same postmortem period. The muscle of the stingray, a marine cartilaginous (elasmobranch) fish, contains the most NPN, according to the findings. When nitrogen concentration was equal, stingray NPN had the strongest hydroxyl radical (OH^●^) and hydrogen peroxide (H_2_O_2_) scavenging activities and metal chelating ability when compared to the others [2]. As a result, antioxidant activity is the principal mechanism of most NPN derived from fish [2]. According to the report [2], NPN is plentiful in stingray muscle and possesses in vitro antioxidant effect. To further validate the antioxidation of stingray NPN, which can be an alternative antioxidant to delay lipid and myoglobin oxidation in dark-fleshed fish and red meat products and thus shelf-life extension, the effects of NPN on lipid and myoglobin oxidations must be investigated. Therefore, the objective of this study was to evaluate the antioxidant activities of NPN from stingray muscle in lecithin liposome and oxymyoglobin model systems to validate its protective role against lipid and myoglobin oxidation after storage for 120 min at varied temperatures (4, 25, and 60 °C).

## 2. Materials and Methods

### 2.1. NPN Fractionation from Stingray Muscle

Stingrays, weighing an average of 0.5 to 1 kg, taken off the coast of Thasala, Nakhon Si Thammarat, Thailand were used. Using the Hashimoto et al. approach [21], NPN was fractionated from stingray muscle. Whole fresh stingray mince was homogenized with 3 vol of iced distilled water (4 °C) at 13,500 rpm for 2 min using an IKA Labortechnik homonenizer (Selangor, Malaysia) in an ice bucket. A Sorvall Model RC-B Plus centrifuge (Newtown, CT, USA) was used to spin the homogenate (5000× *g*/30 min/4 °C). Then, the water-soluble fraction of the supernatant was added along with trichloroacetic acid (TCA) to achieve a final concentration of 5 % (*w*/*v*). The sarcoplasmic protein-containing precipitate was discarded after the dispersion was centrifuged at 5000× *g* (30 min/4 °C). The supernatant was again filtered using Whatman No. 1 filter paper; the filtrate was then collected and given the designation “NPN”. Total nitrogen was measured using the Kjeldahl method [22] after the pH of the NPN solution was brought down to 7.0 using 6 M NaOH. The concentration of NPN in stingray muscle was around 12 mg/kg of wet muscle. In addition, the TCA-soluble peptide and free α-amino acid contents of the NPN were determined [23,24] which were to be 0.28 ± 0.01 µmol tyrosine/g total N and 0.90 ± 0.01 µmol leucine/g total N, respectively.

### 2.2. Effect of Fish NPN on the Oxidative Stability of Lipid in a Lecithin Liposome Model System

Lecithin liposome system was created with some modifications to the approach used by Yi et al. [25]. Soy lecithin (2.4 g) was sonicated for 15 min at an 8 mg/mL concentration in deionized water. NPN solution (10 ppm nitrogen) was added to the lecithin liposome system at various levels (0, 0.5, 1, 5, and 10% (*v*/*v*)) to examine its impact on lipid stability. NPN concentrations ranging from 0 to 10% (*v*/*v*) were added to cover the low to high content, and the concentration was raised proportionally. According to the preliminary test, the maximum concentration was set at 10% (*v*/*v*) due to the prooxidative effect of NPN at higher concentrations. In the assay system for the control, distilled water was added in place of NPN. The reaction mixtures’ final volumes were altered to be equal. The liposome suspensions were again sonicated for 2 min after the addition of NPN. Then, 20 μL of 0.15 M cupric acetate was added to begin the assay. For 120 min in the dark, the mixture was incubated at 4, 25, and 60 °C. To stop the reaction at intervals of 0, 15, 30, 60, and 120 min, liposome sample (1 mL) was added with 0.2% butylated hydroxytoluene (BHT) (20 μL). Liposome oxidation was measured using conjugated diene (CD) [26], peroxide value (PV) [27], and thiobarbituric acid reactive substances (TBARS) [28].

#### 2.2.1. Determination of CD

The absorbance of the liposome sample (0.1 mL) was measured at 234 nm (UV-1900, Shimadzu, Kyoto, Japan) after it was dissolved in 5 mL of methanol [26].

#### 2.2.2. Determination of PV

An organic solvent mixture (chloroform:acetic acid mixture, 2:3, (*v*/*v*)) (25 mL) was used to treat the liposome sample (1.0 g). After giving the mixture a thorough shake, 1 mL of saturated potassium iodide (KI) solution was added. Distilled water (75 mL) was added, and the mixture was mixed after being left in the dark for 5 min. Then, 0.5 mL of starch solution (1%, *w*/*v*) was added to the mixture as an indicator. By titrating the iodine emitted from KI with standardized 0.01 N sodium thiosulfate solution, the PV was ascertained. Milliequivalent of free iodine (meq) per kg of lipid was used to express the PV [27].

#### 2.2.3. Determination of TBARS

The TBARS assay was carried out in accordance with the procedure that Buege and Aust outlined [28]. A solution comprising 0.375% thiobarbituric acid (TBA), 15% trichloroacetic acid, and 0.25 M HCl was added to the liposome sample (0.5 g). After being heated in a boiling water bath (95–100 °C/10 min) to turn pink, the mixture was cooled under running water before being centrifuged at 5500× *g* for 20 min at 25 °C. At 532 nm, the supernatant’s absorbance was measured. The 1,1,3,3-tetramethoxypropane was used at concentrations ranging from 0 to 10 ppm to create a standard curve. The TBARS concentration was determined and represented as mg malondialdehyde (MDA) equivalent/mL liposome.

### 2.3. Effect of Fish NPN on the Redox Stability of Myoglobin in an Oxymyoglobin Model System

Following the procedure outlined by Brown and Mebine [29], oxymyoglobin was prepared from commercial horse heart metmyoglobin. Commercial horse heart metmyoglobin (0.1–0.5 g) was dissolved in 5 mL of 50 mM sodium citrate buffer, pH 6.5. Sodium hydrosulfite was added to the metmyoglobin solution at a ratio of 0.1 mg/mg metmyoglobin and the mixture was vortexed for 10 s. After that, air was bubbled through the metmyoglobin solution using a Pasteur pipette to oxygenate it. The sample was dialyzed for 24 h with 3 changes against 10 L of cold, 50 mM sodium citrate buffer, pH 6.5, to eliminate any remaining sodium hydrosulfite. The concentration of oxymyoglobin was estimated by measuring the absorbance at 525 nm using the millimolar extinction coefficient of 7.6 [29] and adjusting to 2.5 mg/mL. NPN solution (10 ppm nitrogen) was added to the oxymyoglobin solution at various concentrations (0, 0.5, 1, 5, and 10% (*v*/*v*)) to examine its impact on the stability of myoglobin. The reaction mixtures’ final volumes were adjusted to be equal. Then, 120 min were spent incubating the mixture at 4, 25, and 60 °C. The sample was randomly taken for determinations of the contents of oxymyoglobin, metmyoglobin [30], and protein carbonyl [31].

#### 2.3.1. Determination of Oxymyoglobin and Metmyoglobin

The oxidation of oxymyoglobin after 120-min incubation was determined spectrophotometrically by scanning from 650 to 450 nm with a spectrophotometer. The ratio of A630 to A525 and that of A580 to A525 was calculated according to Hansen and Sereika [30]. High A630/A525 and A580/A525 ratios suggest a high relative proportion of metmyoglobin and oxymyoglobin, respectively. The oxymyoglobin remaining and metmyoglobin increased were then calculated in proportion to the initial value (0 min).

#### 2.3.2. Determination of Protein Carbonyl

According to Liu et al. [31], the protein carbonyl content was identified by its reaction with 2,4-dinitrophenylhyrazine (DNPH). Protein carbonylation is the most commonly used measure of oxidative modification of proteins. It is most often measured spectrophotometrically or immunochemically by derivatizing proteins with the classical carbonyl reagent DNPH [31]. A mixture of myoglobin-NPN solution (0.5 mL) and 2.0 mL of 10 mM DNPH in 2 M HCl were combined and left to react for an hour at room temperature (27–29 °C). To precipitate the protein after incubation, 2 mL of 20% TCA was added. The pellet was blow-dried, then dissolved in 1.5 mL of 0.6 M guanidine hydrochloride in 20 mM potassium phosphate buffer (pH 2.3) after being washed twice with 4 mL of ethanol:ethylacetate (1:1, *v*/*v*) combination to remove unreacted DNPH. At 370 nm, protein absorbance was read, and the protein carbonyl content was calculated using a molar absorptivity of 22,400 M^−1^ cm^−1^.

### 2.4. Statistical Analysis

Data were subjected to analysis of variance (ANOVA). Duncan’s multiple range test was used with the statistical analysis program (SPSS 12.0 for Windows, SPSS Inc., Chicago, IL, USA) to identify significant differences between means from triplicate analysis at *p* < 0.05.

## 3. Results and Discussion

### 3.1. CD

**Figure 1:**
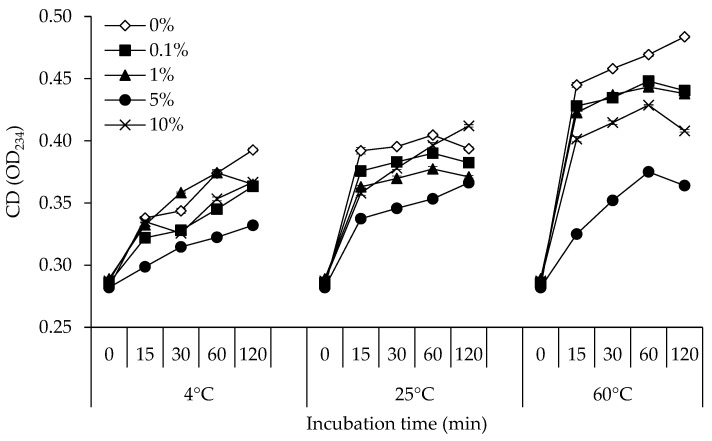
Changes in conjugated diene (CD) of lecithin liposome model system added with 0, 0.1, 1, 5, and 10% (*v*/*v*) of stingray non-protein nitrogen (NPN) incubated at 4, 25 and 60 °C for 0, 15, 30, 60, and 120 min. Bars indicate the standard deviation from triplicate determinations.

Figure 1 displays changes in the CD of a lecithin liposome model system containing 0, 0.1, 1, 5, and 10% of stingray NPN incubated at 4, 25, and 60 °C for 120 min. The lecithin liposome system that was incubated at 4 °C had the lowest CD, which was followed by 25 °C and 60 °C, respectively, in all NPN doses. The CD production was more pronounced at higher storage temperatures. CD significantly increased throughout the incubation period of 60 min (*p* < 0.05), notably in the treatment incubated at 60 °C. Following that, CD tended to fall in all model systems. According to the findings, samples incubated at 60 °C showed the highest rate of CD formation when compared to samples incubated at 25 °C and 4 °C with the same NPN content. There were variable degrees of CD formation and degradation. The amount of CD that accumulates in the lipid fraction may rise if the rate of CD production is higher than the rate of decomposition. Natural unsaturated lipids’ non-conjugated double bonds (C=C-C-C=C) are transformed into conjugated double bonds (C=C-C=C) almost quickly after peroxides are created [32]. According to Nielsen et al. [33], an increase in the TBARS value was closely associated to a decrease in the concentration of CD in meat. From the findings, at all incubation temperatures, the lecithin liposome model system with 5% NPN added tended to have the lowest CD value. The results indicated that, particularly during low temperature storage (4 °C), the addition of 5% NPN could significantly delay the production of CD in a lecithin liposome system.

### 3.2. PV

**Figure 2:**
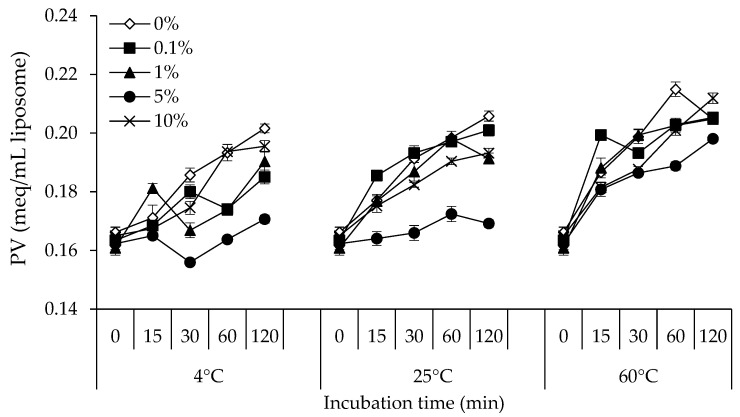
Changes in peroxide value (PV) of lecithin liposome model system added with 0, 0.1, 1, 5, and 10% (*v*/*v*) of stingray non-protein nitrogen (NPN) incubated at 4, 25 and 60 °C for 0, 15, 30, 60, and 120 min. Bars indicate the standard deviation from triplicate determinations.

Unsaturated fatty acids react with molecular oxygen in the complex process of lipid oxidation to produce hydroperoxides, which are the primary oxidation products [32]. PV is typically used to assess the degree of primary lipid oxidation and primarily indicates the amount of hydroperoxides generated during lipid oxidation [34]. Figure 2 displays the PV of the lecithin liposome model system with 0, 0.1, 1, 5, and 10% NPN incubated at 4, 25, and 60 °C for 120 min. The lecithin liposome system that was incubated at 60 °C showed the highest PV, followed by those that were performed at 25 °C and 4 °C, respectively, in all NPN concentrations. With longer incubation times, especially at 60 °C, the PV of the lecithin liposome system tended to rise (*p* < 0.05). Hydroperoxide production was the reason for the increase in PV. When the temperature was raised to 60 °C, the PV of the samples with the same NPN content increased significantly (*p* < 0.05). A balance between the hydroperoxides created and those decomposed during the propagation and termination steps of the oxidation reaction often determines variations in PV [32]. The synthesis and decomposition rates of hydroperoxides may both have been accelerated by increasing temperature, but this effect was more pronounced on the decomposition of hydroperoxides than on their generation [35].

The oxidation of polyunsaturated fatty acids and the interaction of singlet oxygen with unsaturated lipids are two common mechanisms by which lipid hydroperoxides are produced [36]. Some treatments, though, demonstrated decreasing PV with prolonged storage. The secondary oxidation products formed when hydroperoxides decompose were thought to be the cause of the decrease in PV [37]. Aldehydes are among the many decomposition products produced by the multi-step breakdown of hydroperoxides [36]. The findings suggested that NPN compounds from stingray muscle may be able to somewhat inhibit peroxide formation and/or accumulation in the liposome system. At all NPN concentrations tested, the PV tended to increase as the temperature rose. According to the results, the highest PV development was seen at 60 °C and the lowest one at 4 °C. When compared to other concentrations, the lecithin liposome model system supplemented with 5% NPN generally showed the lowest PV, and this inhibitory impact was more obvious during incubation at 4 °C. According to theory, there are three steps to lipid autoxidation: initiation, propagation, and termination. The initiation stage starts with the production of free radicals [38]. According to the findings of the previous investigation [2], NPN from stingray muscle has a disproportionately high capacity to scavenge H_2_O_2_ and OH^●^. This preventative action may involve endogenous peptides and some free amino acids [9]. Stingray NPN may therefore suppress the generation of free radicals and is thought to reduce the creation of primary lipid oxidation products such lipid hydroperoxides when it is delivered in the lecithin liposome system.

### 3.3. TBARS

**Figure 3:**
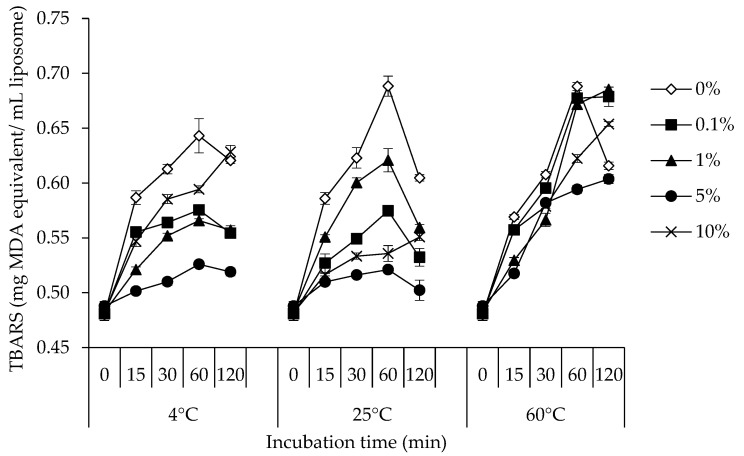
Changes in thiobaribituric acid reactive substances (TBARS) of lecithin liposome model system added with 0, 0.1, 1, 5, and 10% (*v*/*v*) of stingray non-protein nitrogen (NPN) incubated at 4, 25 and 60 °C for 0, 15, 30, 60, and 120 min. Bars indicate the standard deviation from triplicate determinations. MDA = malondialdehyde.

Due to its sensitivity and relatively straightforward approach, the TBARS measurement is the most popular technique for evaluating lipid oxidation in products derived from muscle [39]. The TBA test involves a reaction between TBA and MDA to create a pink complex with a maximum absorbance at 532 nm as a result of the breakdown of lipid hydroperoxide [28]. Figure 3 displays the TBARS values of the lipid in the lecithin liposome model system with 0, 0.1, 1, 5, and 10% of NPN compounds incubated at 4, 25, and 60 °C for 120 min. At the same NPN concentration, the lecithin liposome system incubated at 60 °C had the highest initial TBARS value, followed by those incubated at 25°C and 4 °C, respectively. This demonstrated that, in the lecithin liposome model system, increasing the temperature also had a substantial boosting influence on the generation of secondary lipid oxidation products (*p* < 0.05). The breakdown of hydroperoxides may proceed more quickly as the temperature rises [40].

All lecithin liposome systems experienced a rise in TBARS value during the course of the incubation period of 60 min (*p* < 0.05). Following that, the TBARS levels tended to drop. The significant rise in TBARS was probably brought on by the breakdown of hydroperoxides into secondary oxidation products, particularly aldehydes. The phenomena were amplified as the temperature rose. At the same NPN concentration, the maximum growing TBARS value was recorded at 60 °C and the lowest one at 4 °C. Aldehydes in particular and relatively polar secondary reaction products have been measured using TBARS [32,34]. Nevertheless, some samples showed a lower TBARS value after a longer incubation period. It was hypothesized that the concentration of NPN influenced the antioxidant activity in the lecithin liposome system. However, when compared to other concentrations, the lecithin liposome model system containing 5% NPN demonstrated the lowest growing rate of TBARS value. The lipid oxidation continued to progress even when the NPN concentration was increased to 10%. NPN compounds are substances that can dissolve in water. Excess NPN compounds can dissolve throughout the entire aqueous phase as opposed to deposition at the lecithin liposome system’s interface. As a result, the emulsion became less stable, making the lecithin liposome system more vulnerable to lipid oxidation. Furthermore, the water-soluble prooxidants in NPN, such as non-heme irons, may stimulate lipid oxidation to some extent. The results demonstrated that 5% NPN-containing lecithin liposome model system had a stronger oxidative stability than systems with other NPN concentrations, as seen by the decreased CD, PV, and TBARS values. So, in the lecithin liposome model system, NPN at 5% was a suitable concentration for preventing lipid oxidation.

The majority of amino acids found in the NPN fraction have antioxidant capabilities that are dependent on the pH of the medium and their concentration [41,42]. Free amino acids isolated from seal protein hydrolysate significantly increased oxidative stress at high concentrations while they significantly decreased oxidative stress at low concentrations [43]. Additionally, a variety of aldehydes and hydrocarbons that might be formed from free amino acids and liposomes resulted in the production of numerous characteristics of meat aromas that were distinct from lipid oxidation odor [44]. According to findings from earlier studies, NPN from stingray muscle contained some free amino acids [2]. The majority of amino acids contain antioxidant qualities. Additionally, studies on oil-in-water emulsions have demonstrated that amino acids can prevent lipid oxidation both within the continuous phase and at the interface of emulsion droplets [45]. The findings indicated that the stingray NPN may be able to prevent the development of secondary lipid oxidation products such MDA in a model system using lecithin liposome emulsions.

### 3.4. Myoglobin Oxidation

**Figure 4:**
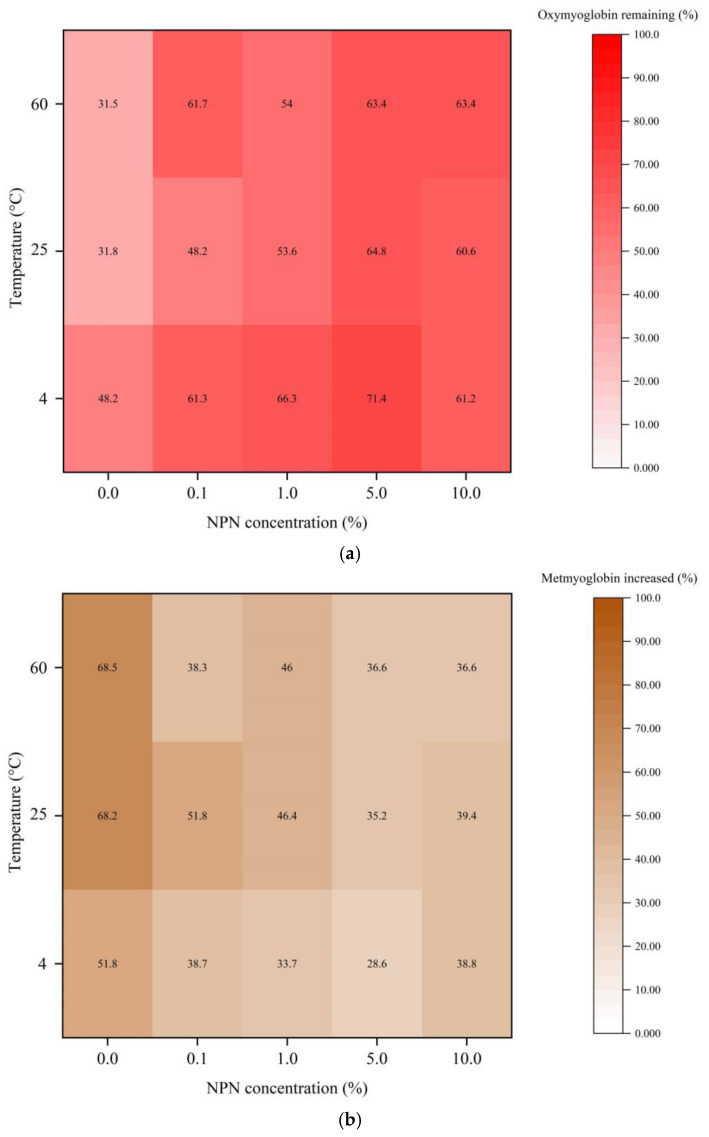
Heat maps of the remaining oxymyoglobin (**a**) and increased metmyoglobin (**b**) of the oxymyoglobin model system added with 0, 0.1, 1, 5, and 10% (*v*/*v*) of stingray non-protein nitrogen (NPN) incubated at 4, 25, and 60 °C for 120 min. The remaining oxymyoglobin and increased metmyoglobin were calculated in proportion to the initial value (0 min).

**Figure 5:**
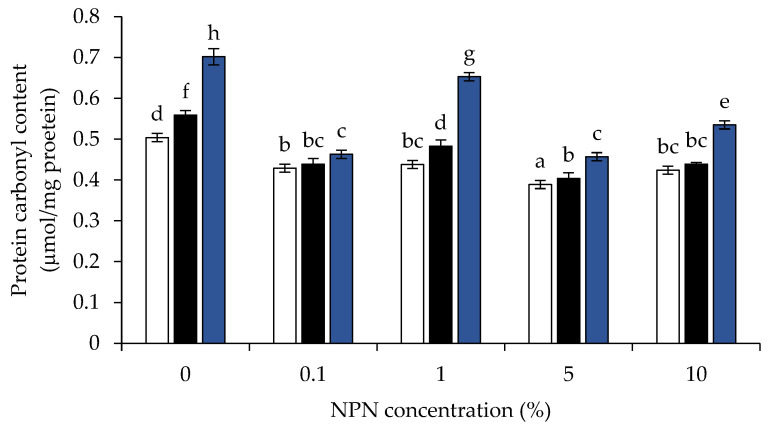
Changes in the protein carbonyl content of oxymyoglobin model system added with 0, 0.1, 1, 5, and 10% (*v*/*v*) of stingray non-protein nitrogen (NPN) incubated at 4 °C (white), 25 °C (black), and 60 °C (blue) for 120 min. The initial carbonyl content for all treatments was 0.324 μmol/mg protein. Bars represent standard deviation from triplicate determinations. Different letters (a–h) indicate significant differences (*p* < 0.05).

Figure 4 and Figure 5 demonstrate, respectively, the percentages of oxymyoglobin remaining and metmyoglobin increased from a myoglobin model system containing 0, 0.1, 1, 5, and 10% stingray NPN compounds incubated at 4, 25, and 60 °C for 120 min. At all incubation temperatures, there was no difference between the initial (0 min) contents of oxymyoglobin and metmyoglobin in any of the model systems (*p* > 0.05). In both model systems, oxymyoglobin content dropped with longer incubation times whereas metmyoglobin production rose. Myoglobin may have undergone greater oxidation as seen by the rise in metmyoglobin concentration with increased storage duration. When incubated at a low temperature (4 °C), oxymyoglobin in the model system tended to be more stable than when maintained at high temperatures (25 °C and 60 °C, respectively). As a result, it was discovered that the model systems incubated at 4 °C formed less metmyoglobin than those incubated at 25 °C and 60 °C, respectively. Higher temperatures would more effectively release non-heme iron by causing the globin moiety in myoglobin molecules to denature [46]. As a result, myoglobin oxidation was seen to occur more quickly the higher the applied temperature. It was discovered that NPNs’ ability to prevent myoglobin oxidation was concentration-dependent when taking that impact into account. Myoglobin undergoes oxidation to produce metmyoglobin, which involves the conversion of iron from its ferrous state to its ferric state. Free radicals such as the OH^●^ and H_2_O_2_ may promote this process [47]. According to findings from earlier studies [2], NPN from stingray muscle had a disproportionately high capacity to scavenge H_2_O_2_ and OH^●^. As a result, the oxidation of myoglobin in the model system may be decreased by NPN from stingray muscle. Based on the results, when compared to other NPN concentrations, the presence of 5% NPN exhibited the lowest rate of oxymyoglobin oxidation and the generation of metmyoglobin. Oxymyoglobin oxidation was higher at 10% NPN, notably at 4 and 25 °C, than at 5% NPN. This was most likely owing to the presence of water-soluble pro-oxidants such as soluble non-heme iron in higher concentrations in the NPN fraction, which can speed up the oxidation of oxymyoglobin. Thus, the oxidation of myoglobin could be suppressed by NPN at 5% more efficiently than at other concentrations. Additionally, combining low temperature (4 °C) storage with stingray NPN improved their ability to inhibit myoglobin oxidation.

### 3.5. Protein Carbonyl Content

One of the most dramatic chemical changes to oxidized proteins is protein carbonylation, which is widely acknowledged [31]. Figure 5 displays the protein carbonyl concentration of myoglobin in a myoglobin model system comprising 0, 0.1, 1, 5, and 10% of stingray NPN compounds incubated at 4, 25, and 60 °C for 120 min. At all incubation temperatures there was no difference in the initial (0 min) carbonyl content of any model systems (*p* > 0.05). All model systems showed an increase in carbonyl content when incubation duration was increased. Protein may have undergone greater oxidation as seen by the rise in carbonyl concentration with increased storage duration. Particularly when adding with NPN to myoglobin model system at 1, 5, and 10%, the carbonyl concentration in model system incubated at low temperature (4 °C) tended to persist more than at high temperatures (25 °C and 60 °C, respectively) (Figure 5). It was discovered that the capacity to prevent protein oxidation was concentration-dependent when taking into account NPNs’ protective impact against it (Figure 5). According to the findings, when 5% NPN was present, the rate of protein oxidation was the lowest when compared to other NPN concentrations during the course of the incubation period. NPN at 1% may drive the darkening of oxymyoglobin solution via oxidation, as evidenced by the high degree of metmyoglobin production (Figure 4b), as well as via the amine-carbonyl Maillard reaction at 60 °C. NPN at 1% concentration may be the optimal concentration for the Maillard reaction in this system. As a result, the protein carbonyl content was higher at 60 °C in the presence of 1% NPN than at other NPN concentrations. Protein carbonyl was larger at 10% NPN than at 5% NPN due to free iron aided protein oxidation due to the presence of higher non-heme iron content, as previously discussed in both lecithin liposome and oxymyoglobin model systems.

The outcome suggested that NPNs at 5% could more effectively control protein oxidation than at other doses. Additionally, combining low temperature (4 °C) storage with stingray NPN improved their ability to inhibit protein oxidation. The sample with the highest levels of protein oxidation would also have the highest levels of metmyoglobin production. According to Promeyrat et al. [48], the conversion of oxymyoglobin to metmyoglobin produces the OH^●^, which dismutates into H_2_O_2_. Myoglobin, a naturally occurring component of muscle, has been shown to increase protein oxidation and, more specifically, protein carbonylation [49]. Metmyoglobin encouraged the synthesis of carbonyls to a larger extent than a metal-catalyzed oxidizing system (Fe^3+^/ascorbic acid/H_2_O_2_), according to Park et al. [50]. H_2_O_2_ can also cause the release of iron from heme molecules, and the non-heme iron subsequently catalyzes oxidative processes [51].

## 4. Conclusions

Lecithin liposome and myoglobin solution model systems were employed to evaluate the impact of stingray NPN (10 ppm nitrogen) on the oxidative stability of lipid and myoglobin. At all incubation temperatures, lecithin liposome and myoglobin solution supplemented with 5% (*v*/*v*) NPN tended to demonstrate the lowest levels of lipid and myoglobin oxidation. As a result, a stingray NPN solution at 5% (*v*/*v*) might be utilized as a natural antioxidant to prevent lipid and myoglobin from oxidation while being incubated at a low temperature (4 °C).

## Data Availability

Data is contained within the article.
